# The Early Expression of HLA-DR and CD64 Myeloid Markers Is Specifically Compartmentalized in the Blood and Lungs of Patients with Septic Shock

**DOI:** 10.1155/2016/3074902

**Published:** 2016-06-19

**Authors:** Tomasz Skirecki, Małgorzata Mikaszewska-Sokolewicz, Grażyna Hoser, Urszula Zielińska-Borkowska

**Affiliations:** ^1^Nalecz Institute of Biocybernetics and Biomedical Engineering, Polish Academy of Sciences, 4 Trojdena Street, 02-109 Warsaw, Poland; ^2^Department of Anesthesiology and Intensive Care Medicine, The Center of Postgraduate Medical Education, Czerniakowska 231 Street, 00-416 Warsaw, Poland; ^3^The 1st Clinic of Anesthesiology and Intensive Care Medicine, The Medical University of Warsaw, Lindleya 4 Street, 02-005 Warsaw, Poland

## Abstract

Identification of reliable biomarkers is key to guide targeted therapies in septic patients. Expression monitoring of monocyte HLA-DR and neutrophil CD64 could fulfill the above need. However, it is unknown whether their expression on circulating cells reflects the status of tissue resident cells. We compared expressions of HLA-DR and CD64 markers in the circulation and airways of septic shock patients and evaluated their outcome prognostic value. The expression of CD64 on neutrophils and HLA-DR on monocytes was analyzed in the peripheral blood and mini-bronchoalveolar lavage fluid cells by flow cytometry. Twenty-seven patients with septic shock were enrolled into the study. The fluorescence intensity of HLA-DR on circulating monocytes was 3.5-fold lower than on the pulmonary monocytes (*p* = 0.01). The expression of CD64 on circulating and airway neutrophils was similar (*p* = 0.47). Only the expression of CD64 on circulating neutrophils was higher in nonsurvivors versus survivors (2.8-fold; *p* = 0.031). Pulmonary monocytes display a higher level of HLA-DR activation compared to peripheral blood monocytes but the expression of neutrophil CD64 is similar on lung and circulating cells. Death in septic patients was effectively predicted by neutrophil CD64 but not monocytic HLA-DR. Prognostic value of cellular activation markers in septic shock appears to strongly depend on their level of compartmentalization.

## 1. Introduction

Severe sepsis and septic shock remain major cause of deaths in the intensive care units (ICUs) around the globe [1]. Yet, to date, early diagnosis, aggressive antibiotic treatment, and nonspecific therapeutic procedures recommended by the Sepsis Surviving Campaign Guidelines constitute the only evidence-based available treatments [1].

The immune response plays central pathophysiological role in the development and progression of sepsis. In spite of numerous clinical trials with agents modifying the host response during sepsis, almost all have failed to show efficiency [2]. Undoubtedly, the insufficient understanding of the immune response in sepsis as well as lack of the immune status-based stratification prior to various treatments belongs the major reasons of those trial failures. Therefore, establishment of accurate biomarkers of immune response during sepsis is crucial. Since sepsis has been until very recently defined as a host's systemic inflammatory response to infection, it is often incorrectly assumed that the immune changes in sepsis are generally unified throughout the entire organism. However, many indications suggest a strong compartmentalization of the host's immune responses [3]. For example, Cavaillon's group reported varying endotoxin tolerance of macrophage derived from different sites of LPS-treated mice [4]. Consequently, a concept of “compartmentalization” of inflammatory response during sepsis has been proposed [5]. Unfortunately, confirmatory data from human studies are lacking due to obvious investigative limitations. Peripheral blood (PB) is the most commonly used and valuable diagnostic probe in the clinical setting. However, most of the immune reactions in the body occur beyond the PB in sites such as lymph nodes, spleen, and inflamed tissues that are typically beyond reach of standard diagnostics.

A molecule that has gained major attention as an immunosuppression biomarker in sepsis is the human leukocyte antigen-DR (HLA-DR) constitutively expressed by monocytes. Diminished expression of HLA-DR on blood monocytes has been shown to accurately predict septic complications in trauma, surgery, and burns [6–8]. Low HLA-DR correlated with nosocomial infections after septic shock and in some studies with survival [9]. Monocytes are the key part of the early response to infection and easily migrate from the circulation into inflamed/pathogen-invaded tissues [10]. However, the status of the tissue-located monocytes has never been studied in human sepsis.

CD64 is the only human high-affinity receptor for IgG (Fc*γ*RI) and, under steady-state conditions, it is expressed mainly by monocytes/macrophages [11]. However, during infections, it is also robustly upregulated on the neutrophils [12]. This phenomenon was a base for several studies using the CD64 expression as a marker of bacteremia and sepsis, especially in neonates [13]. Most of the reports have positively verified its utility as a valuable biomarker for distinguishing sepsis from other SIRS-related conditions [14].

Most of the existing human studies examined the HLA-DR and CD64 on cells from the peripheral blood only. During sepsis of various origins, an increased migration of monocytes and neutrophils into the lungs can be observed [10]. While potentially beneficial during pneumonia, this response can be harmful and contribute to the development of sepsis-induced acute lung injury [15].

Given the above, we hypothesized that in patients with septic shock neutrophils and monocytes in the airways and alveoli differ from those present in the circulation. We designed a prospective observational study aiming to (1) compare their expression on cells from peripheral blood and bronchoalveolar lavage fluid (BAL) and (2) evaluate the utility of monitoring CD64 expression on neutrophils and HLA-DR on monocytes as prognostic markers in septic shock patients.

## 2. Methods

### 2.1. Patients

This study was performed in two teaching hospital general ICUs (Professor Orlowski Independent Public Clinical Hospital and The Infant Jesus Teaching Hospital in Warsaw). Patients with suspected or proved infection who fulfilled the criteria of septic shock according to the ACCP/SCCM Consensus Conference definitions [16] were enrolled into the study within 24 hours after diagnosis over a period between April 2012 and January 2014. The exclusion criteria included age <18 or >85 years, pregnancy, immunosuppressive treatment, innate or acquired immunodeficiency, and disseminated cancer. All consecutive patients were screened for the availability for the study. All patients were receiving treatment according to the Surviving Sepsis Campaign Guidelines 2012 [17]. Routine microbiological, biochemical, and imaging tests were performed and clinical severity scores were calculated. The study was approved by the institute's Ethic Board and patients informed consent was achieved in agreement with the rules of the Ethic Committee.

### 2.2. Sample Collection

Peripheral blood samples were obtained via arterial catheter. One milliliter of PB was placed into tubes with EDTA anticoagulant. Bronchoalveolar lavage fluid (BALf) was collected with mini-BAL technique (2x 20 mL wash with saline). Probes were analyzed within 3 hours from collection. BALf was treated according to the guidelines of Polish Society of Lung Diseases [18]. Briefly, the fluid was filtered through sterile gauze, centrifuged, and resuspended in PBS. White blood count (WBC) was calculated using Bürker's hemocytometer and Türk's solution. Due to logistic reasons mini-BAL was collected from 20 patients.

### 2.3. Flow Cytometry

**Figure 1:**
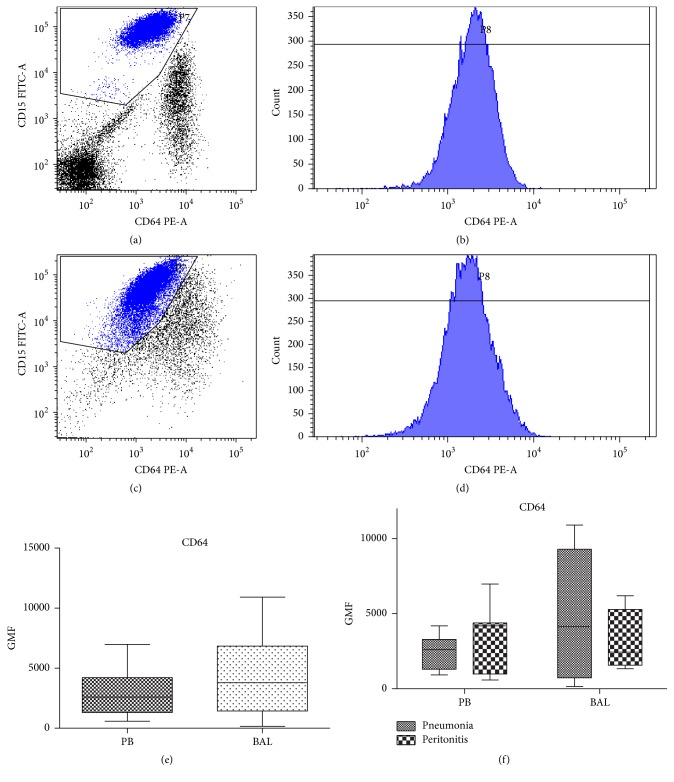
Expression of the CD64 on neutrophils from septic shock patients. Gating of the CD15+ granulocytes on the CD15 versus CD64 dot plot in peripheral blood (a) and BALf (c). Expression of CD64 on the gated CD15+ neutrophils from peripheral blood (b) and BALf (d). Comparison of the geometric median fluorescence of the CD64 on CD15+CD64med neutrophils from peripheral blood (PB) and bronchoalveolar lavage (BAL) from septic shock patients (e). Comparison between BAL and PB monocytes in patients with pneumonia and peritonitis (*n* = 20) (f).

Immunocytochemical staining was performed as described [19]. Briefly, 60 *μ*L of PB or BAL resuspended cells was stained with 10 *μ*L of anti-CD64 PE antibody and 5 *μ*L of anti-CD15 FITC antibody or 5 *μ*L of anti-CD14 PerCP and anti-HLA-DR FITC (all BD Biosciences, San Jose, CA, USA) for 25 min in room temperature. Then cells were lysed with BD Pharm Lyse for 10 minutes, centrifuged, washed with PBS, and fixed in 0.5% paraformaldehyde in PBS (Sigma Aldrich, St. Louis, USA). Cells were analyzed using FACS Canto II flow cytometer with FACS Diva software (BD, San Jose, CA, USA). A hundred thousand events were recorded. To standardize the analysis we applied QuantiBRITE Phycoerythrin Beads (BD Biosciences, San Jose, CA, USA). These beads enable reliable quantitation of the antibodies bound per cell (ABC), a value that is standardized and can be easily compared between instruments and laboratories [20]. Beads were used according to the manufacturer's protocol. Reconstituted beads were acquired with the same instrument settings as for the cellular analysis. For the analysis of the level of CD64 expression specifically on neutrophils, cells were gated on the morphological SSC/FCS dot blot and then plotted on the CD15 versus CD64 cytogram (Figure 1). Polygonal gate for neutrophils was drawn on CD15+ and CD64 negative/dim/positive cells. CD64hiCD15neg/low cells were considered monocyte/macrophage.

### 2.4. Statistical Analyses

All data are expressed as median and P_25_–P_75_ percentiles. Comparisons between groups were performed with Mann-Whitney *U* test. The regression equation of geometric mean fluorescence of CD64 was used to calculate the ABCs. CD64 ABCs from PB and BALf from the same patients were compared using Wilcoxon's test for paired samples. Fisher's exact test was used for comparisons of dichotomous variables. Receiver operating characteristic (ROC) curves were constructed for CD64 expression and areas under curve (AUCs) were calculated to assess the prognostic performance of the test. Sensitivity, specificity, positive predictive value (PPV), and negative predictive value (NPV) were calculated for cut-off values selected following ROC analysis. *p* < 0.05 was considered significant. Statistica 10.0 and GraphPad Prism 5 software were used for the data analysis and preparing graphs, respectively.

## 3. Results

### 3.1. Patient Characteristics

Twenty-seven adult patients with septic shock, within 24 hours of its onset, were enrolled into the study; eight patients who fulfilled the entry criteria had to be (randomly) excluded due to the technical lack of possibility to perform FACS analysis. The characteristics of the patients are summarized in Table 1. Median age of the patient's population was 61 and the most common causes of septic shock were peritonitis (60%) and pneumonia (33%). The high severity of the septic shock was reflected by the ICU morality rate that reached 60%. BAL was obtained from 20 of these patients. In this group the median age was 57, the group included 12 women (60%), and the mortality was 55%.

### 3.2. CD64 Expression on Peripheral Blood and Airway Neutrophils from Septic Patients

For a better discrimination of granulocytes in the flow cytometric analysis, we set the gate for neutrophils on CD64/CD15 dot plot (Figure 1(a)). Median frequency of CD15+ granulocytes in the peripheral blood was 70.6%. The median GMF for CD64 on peripheral blood neutrophils was 2 683 (1466–4402; 25–75 percentile). These values were not significantly different for the lung-resident neutrophils, in which CD64 median expression was 3 782 (1 435–6 830) (Figure 1(b), *n* = 20; *p* = 0.4648). No differences between circulating and lung-resident neutrophils were apparent when the values for CD64 expression for patients with peritonitis and pneumonia were analyzed separately (Figure 1(d)).

### 3.3. HLA-DR Expression on Peripheral Blood and Airway Monocytes from Septic Patients

**Figure 2:**
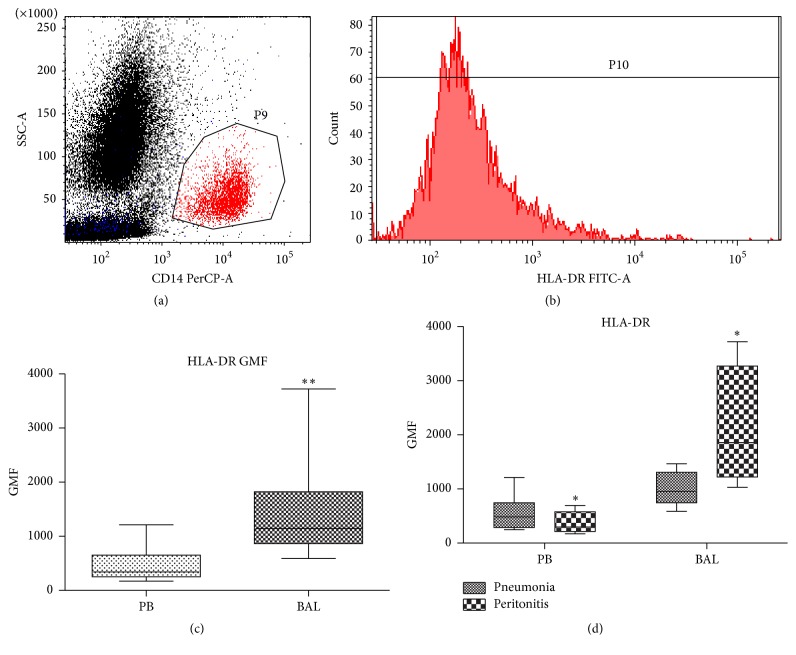
Expression of HLA-DR on monocytes from septic shock patients. GMF geometric median fluorescence. (a) Gating of the CD14+ monocytes on the side scatter characteristic (SSC) versus CD14 dot plot. (b) Expression of HLA-DR on the gated CD14+ monocytes. (c) Comparison of the geometric median fluorescence of the HLA-DR on CD14+ monocytes from peripheral blood (PB) and bronchoalveolar lavage (BAL) from septic patients. (d) Comparison between BAL and PB monocytes in patients with pneumonia and peritonitis (*n* = 20). ^*∗*^ *p* < 0.05; ^*∗∗*^ *p* < 0.01.

CD14-positive monocytes constituted 3.8% (2.4–5.9) of the white blood count of septic patients. Their median expression of HLA-DR was 342 GMF (252–652) and it was 3.5-fold lower (*p* = 0.01) in comparison to the pulmonary monocytes in which median expression of HLA-DR was 3.5-fold higher (*n* = 20, *p* = 0.0098) (Table 2, Figure 2). In patients with pneumonia-sepsis, the HLA-DR expression was 2-fold higher on pulmonary monocytes comparing to those in the peripheral blood (Table 2, *p* = 0.1563). Patients with peritonitis had 7-fold higher expression of HLA-DR on pulmonary monocytes in comparison to those in circulation (*p* = 0.068, Figure 2(d)).

### 3.4. CD64 Expression on Neutrophils but Not HLA-DR Expression on Monocytes Predicted Mortality in Septic Patients

HLA-DR expression on the peripheral blood monocytes did not vary between survivors and nonsurvivors (Table 1). In contrast, the expression of CD64 on the circulating neutrophils was 2.8-fold higher in the patients that died during the hospital stay (*p* = 0.03). Expression of CD64 was also normalized as antibodies bound per cell (ABC) value using the QuantiBRITE system. The median ABC of circulating neutrophils was 28.5 × 10^6^ (11.7 × 10^6^–58.9 × 10^6^). ROC analysis was applied to assess the prognostic performance of neutrophil CD64 ABC to predict mortality. The AUC for CD64 ABC on circulating neutrophils on day 1 (of septic shock diagnosis) was 0.86 (95% CI 0.69–1.04). The cut-off ABC value selected with Youden's Index (0.77) was 27 117 912 and it displayed sensitivity of 77% and specificity of 100%. Characteristics of selected cut-off values are presented in Table 3.

## 4. Discussion

There is an urgent need for identifying biomarkers that would enable proper monitoring of the immune status in septic patients, a prerequisite to successfully guide any immunomodulatory treatment. Numerous cytokines, mediators, cells, and cell-related molecules have been proposed as candidate biomarker [21] but only a handful appears as meaningful.

In this study, we analyzed the expression of activation markers of two types of myeloid cells in the peripheral blood and lungs of septic patients, simultaneously. We also measured the expression of HLA-DR on pulmonary monocytes retrieved by mini-BAL. Even though not ideal, this technique is rapid and safe for analysis of airspace-resident cells [22, 23]. SSC/CD14 FACS gating enabled us to distinguish monocytes from alveolar macrophages that do not express CD14 [24]. Interestingly, HLA-DR expression was 3.5-fold higher on pulmonary monocytes compared to those from peripheral blood (Figure 2). Surprisingly, this difference was most pronounced in the subgroup of patients with septic shock from peritonitis (not pneumonia). This novel observation indicates that the difference in the compartment-specific HLA-DR expression is not simply a result of stronger local activation of monocytes at the site of primary infection, but rather a global effect. Moreover, it strongly implies a dissimilar immune response in sepsis syndromes of different origin previously suggested by our group and others [19, 25]. Peritonitis-induced lung trafficking of monocytes has been described in the murine model of intraperitoneal endotoxin injection [26]. Monocytes recruited to inflamed murine lungs expressed highly distinct genes in comparison to the steady-state lung-resident mononuclear phagocytes [27]. Cavaillon's group showed that only alveolar cells are resistant to endotoxin tolerance in mouse endotoxemia [4]. They also revealed that lung-resident B cells maintain (via secretion of IL-18 and IFN-*γ*) the endotoxin sensitivity of alveolar macrophages [28]. Limited human data are contradictory; using the same dose of endotoxin, human alveolar cells either did [29] or did not produce TNF alpha [30] upon intratracheal challenge. Direct instillation of endotoxin into the lungs of human volunteers stimulated production of TNF alpha, IL-1*β*, and IL-6 [31]. Conversely, alveolar macrophages from patients with severe pneumonia displayed upregulated transcripts for IL-8, but not TNF and IL-6 [32]. Those observed discrepancies were likely due to the different study setups, for example, nonuniform models, various time of cell collection, and potential endotoxin contamination. Although this study was not designed to compare lung monocytes from healthy controls and septic patients, our results strongly indicate compartment-dependent differences in monocytes from septic patients. As the expression of HLA-DR on monocytes has been shown to correlate with their functional capacities [33, 34], we hypothesize that pulmonary monocytes are maintained at higher activation status despite strong suppression of those in the systemic circulation.

When we compared the expression of CD64 on the PB neutrophils with those from BAL fluid, no differences were apparent. This finding sharply contrasts the compartment-related differences of monocyte HLA-DR expression. However, our observation agrees with another study that demonstrated a significant correlation of inflammatory response between* ex vivo* LPS-treated PB neutrophils and alveolar neutrophils upon LPS instillation into the lungs of human volunteers [35]. Despite this report, our results were not obvious. Differences in the patterns of cytokine production by airway and circulating neutrophils were reported [36, 37]. Unfortunately, those two studies did not evaluate the phenotype markers of the neutrophils; both assessed patients with chronic inflammatory conditions, which is in contrast to the acutely ill septic patients (on the first day of the diagnosis) in the current study. Therefore, it is possible that the described differences in the neutrophil activity may be attributable to the chronic inflammation.

We have also evaluated the predictive value of the expression of neutrophil CD64 and monocyte HLA-DR in septic patients. In our cohort of patients, day 1 expression of HLA-DR on the peripheral blood monocytes did not differ between survivors and nonsurvivors. This finding in not surprising as the monitoring of HLA-DR was primarily used for the prediction of sepsis development following other clinical preexisting conditions such as surgery, burns, and trauma [6–8, 38]. Similarly, some studies showed poor correlation with prognosis [9], while others indicated that kinetics of the HLA-DR expression over the time can serve as a marker of prognosis [39, 40].

In our study, the expression of CD64 on blood neutrophils was 2.8-fold higher in the nonsurviving patients (Table 1). We calculated the CD64 expression as antibodies bound per cell (ABC) in order to enable comparison of our results with future studies on the utility of this marker in septic shock prognosis. Not only was the prognostic performance of neutrophil CD64 in our cohort of patients very good (AUC = 0.86) but also its specificity and positive predictive value were high, potentially enabling a precise identification of patients at the high risk of death. Most of the previous studies on CD64 were focused on the diagnostic use of this marker to distinguish infection and sepsis from other SIRS conditions [13]. The utility of neutrophil CD64 as a prognostic marker in sepsis is unclear. Three studies corroborate our findings by demonstrating correlation between the CD64 expression and sepsis mortality [41–43]. In contrast, Danikas et al. reported a lack of such relationship [44]. This contradiction might have been influenced by several factors including dissimilar makeup of studied populations or timing of samples collection. Aside from this, discrepancies regarding correlation of the CD64 expression with the functional capacity of the examined neutrophils exist. For example, correlation changes between neutrophil CD64 expression and their phagocytic capacity [44] as well as their respiratory burst activity were shown [45]. Contradictory conclusions were drawn by Hirsh et al. [46]. Although we did not assess the phagocytic activity of neutrophils, our results are in agreement with those suggesting functional impairment due to upregulation of CD64 that in turn leads to impairment of antibacterial properties.

Given the above, we hypothesize that the observed differences between the prognostic performance of monocytic HLA-DR and neutrophilic CD64 in septic patients may be due to the different compartmentalization of these markers. CD64 is expressed on the similar level in the peripheral blood and airway neutrophils, irrespective of the primary infection site. On the contrary, the HLA-DR expression on lung monocytes is much higher in comparison to the circulating monocytes and this difference predominates in the peritonitis patients. Conversely, markers expression which is less compartment-specific is expected to have a better prognostic value. If verified in subsequent studies, our observation poses serious diagnostic and therapeutic implications.

Our study has several limitations. First, we enrolled a homogenous group of severely ill patients with high mortality risk; the performance of prognostic markers can differ in other (e.g., less severe) patient cohorts. Second, the size of the examined group was limited and our findings should be reconfirmed in a bigger population. Last, we assessed only phenotypic markers of the cells but not their functionality. The current results indicate that examination of the functional capacity of PB and BAL cells is necessary to obtain a deeper insight into the aforedescribed relationship.

Summarizing, our study revealed a distinct compartmentalization of commonly studied markers of immune activation in sepsis: CD64 on neutrophils and HLA-DR on monocytes. Monocytic HLA-DR, marker whose expression is highly compartment-dependent, demonstrated a poor prognostic performance. It is suggestive that analysis of blood monocytes does not reflect the status of resident/migrating monocytes in other tissues and may be diagnostically flawed. Conversely, neutrophil CD64, whose expression was similar in the circulating and lung-resident cells, showed a good prognostic accuracy when measured in the blood. It can be, therefore, assumed that CD64 expression on the circulating cells reflects their systemic status. Overall, we suggest that assessment of immune markers which are highly compartmentalized in septic shock may be misleading in prognosis-making and/or guidance of a immunomodulatory treatment.

## Figures and Tables

**Table 1:** Clinical characterization of the studied group.

Characteristics	Survivors (*n* = 11)	Nonsurvivors (*n* = 16)	*p*
Age	66 (41–75)	55 (52–83)	0.7586
Female (*n*)	5	10	0.3811
SOFA	5 (4–10)	12 (8–12)	**0.0022**
Source of infection (*n*)			
*Peritonitis*	7	9	
*Pneumonia*	2	7	
*Other*	2	0	
Mini-BAL samples (*n*)	9	11	
PB CD64 GMF	1 208 (864–1 743)	3 428 (1 772–4 369)	**0.0313**
PB HLA-DR GMF	347 (251–473)	342 (243–692)	1.0000

Results are presented as median and P_25_–P_74_ percentiles.

**Table 2:** Expression of myeloid markers on circulating and pulmonary monocytes and neutrophils (*n* = 20).

Parameter	Peripheral blood	Lung	*p*
CD64 GMF	2 683 (1466–4402)	3 782 (1 435–6 830)	0.4648
*Pneumonia*	2 055 (1 209–2 818)	6 830 (793–9 831)	0.1875
*Peritonitis*	4 192 (982–4386)	2 447 (1 579–5 287)	0.1250
HLA-DR GMF	342 (252–652)	1144 (889–1789)	**0.0098**
*Pneumonia*	484 (288–746)	953 (743–1309)	**0.0138**
*Peritonitis*	298 (216–579)	1858 (1221–3272)	0.0678

GMF: geometric mean fluorescence; PB: peripheral blood.

**Table 3:** Prognostic performance for selected values of ABC of CD64 on circulating neutrophil on day 1 of the diagnosis of septic shock.

CD64 D1 (ABC)	Sensitivity (%)	Specificity (%)	PPV (%)	NPV (%)
27 117 912	77	100	100	67
17 011 428	77	83	91	63
14 570 676	77	80	91	57

ABC: antibodies bound per cell; NPV: negative predictive value; PPV: positive predictive value.

## Abbreviations

ABC: Antibodies per cell
ACCP: American College of Chest Physicians
AUC: Area under curve
BAL: Bronchoalveolar lavage
CD64: Cluster of differentiation 64
FSC: Forward-scattered light
GMF: Geometric mean fluorescence
HLA-DR: Human leukocyte antigen-DR
ICU: Intensive care unit
NPV: Negative predictive value
PB: Peripheral blood
PE: Phycoerythrin
PerCP: Peridinin-chlorophyll proteins
PPV: Positive predictive value
ROC: Receiver operating characteristic
SIRS: Systemic inflammatory response
SCCM: Society of Critical Care Medicine
SSC: Side-scattered light
WBC: White blood cells.

## References

[1] Levy M. M., Artigas A., Phillips G. S. (2012). Outcomes of the surviving sepsis campaign in intensive care units in the USA and Europe: a prospective cohort study. *The Lancet Infectious Diseases*.

[2] Cohen J., Vincent J.-L., Adhikari N. K. J. (2015). Sepsis: a roadmap for future research. *The Lancet Infectious Diseases*.

[3] Schwacha M. G., Schneider C. P., Chaudry I. H. (2002). Differential expression and tissue compartmentalization of the inflammatory response following thermal injury. *Cytokine*.

[4] Fitting C., Dhawan S., Cavaillon J.-M. (2004). Compartmentalization of tolerance to endotoxin. *Journal of Infectious Diseases*.

[5] Cavaillon J.-M., Annane D. (2006). Compartmentalization of the inflammatory response in sepsis and SIRS. *Journal of Endotoxin Research*.

[6] Cheadle W. G., Hershman M. J., Wellhausen S. R., Polk H. C. (1991). HLA-DR antigen expression on peripheral blood monocytes correlates with surgical infection. *The American Journal of Surgery*.

[7] Hershman M. J., Cheadle W. G., Wellhausen S. R., Davidson P. F., Polk H. C. (1990). Monocyte HLA-DR antigen expression characterizes clinical outcome in the trauma patient. *British Journal of Surgery*.

[8] Venet F., Tissot S., Debard A.-L. (2007). Decreased monocyte human leukocyte antigen-DR expression after severe burn injury: correlation with severity and secondary septic shock. *Critical Care Medicine*.

[9] Lukaszewicz A.-C., Grienay M., Resche-Rigon M. (2009). Monocytic HLA-DR expression in intensive care patients: interest for prognosis and secondary infection prediction. *Critical Care Medicine*.

[10] Yin K., Wilmanski J., Wang C., Qiu G., Tahamont M. (2000). Lung compartmentalization of inflammatory cells in sepsis. *Inflammation*.

[11] Mancardi D. A., Albanesi M., Jönsson F. (2013). The high-affinity human IgG receptor Fc*γ*RI (CD64) promotes IgG-mediated inflammation, anaphylaxis, and antitumor immunotherapy. *Blood*.

[12] Leino L., Sorvajärvi K., Katajisto J. (1997). Febrile infection changes the expression of IgG Fc receptors and complement receptors in human neutrophils in vivo. *Clinical and Experimental Immunology*.

[13] Bhandari V., Wang C., Rinder C., Rinder H. (2008). Hematologic profile of sepsis in neonates: neutrophil CD64 as a diagnostic marker. *Pediatrics*.

[14] Cid J., Aguinaco R., Sánchez R., García-Pardo G., Llorente A. (2010). Neutrophil CD64 expression as marker of bacterial infection: a systematic review and meta-analysis. *Journal of Infection*.

[15] O'Dea K. P., Wilson M. R., Dokpesi J. O. (2009). Mobilization and margination of bone marrow Gr-1high monocytes during subclinical endotoxemia predisposes the lungs toward acute injury. *The Journal of Immunology*.

[16] Levy M. M., Fink M. P., Marshall J. C. (2003). SCCM/ESICM/ACCP/ATS/SIS international sepsis definitions conference. *Critical Care Medicine*.

[17] Dellinger R. P., Levy M. M., Rhodes A. (2013). Surviving sepsis campaign: international guidelines for management of severe sepsis and septic shock: 2012. *Critical Care Medicine*.

[18] Chcialowski A., Chorostowska-Wynimko J., Fal A., Pawlowicz R., Domagala-Kulawik J. (2011). Recommendation of the Polish Respiratory Society for bronchoalveolar lavage (BAL) sampling, processing and analysis methods. *Pneumologia i Alergologia Polska*.

[19] Hoser G. A., Skirecki T., Złotorowicz M., Zielińska-Borkowska U., Kawiak J. (2012). Absolute counts of peripheral blood leukocyte subpopulations in intraabdominal sepsis and pneumonia-derived sepsis: a pilot study. *Folia Histochemica et Cytobiologica*.

[20] Pannu K. K., Joe E. T., Iyer S. B. (2001). Performance evaluation of quantiBRITE phycoerythrin beads. *Cytometry*.

[21] Skirecki T., Borkowska-Zielińska U., Złotorowicz M., Hoser G. (2012). Sepsis immunopathology: perspectives of monitoring and modulation of the immune disturbances. *Archivum Immunologiae et Therapiae Experimentalis*.

[22] Colucci G., Domenighetti G., Della Bruna R. (2009). Comparison of two non-bronchoscopic methods for evaluating inflammation in patients with acute hypoxaemic respiratory failure. *Critical Care*.

[23] Pace E., Giarratano A., Ferraro M. (2011). TLR4 upregulation underpins airway neutrophilia in smokers with chronic obstructive pulmonary disease and acute respiratory failure. *Human Immunology*.

[24] Wahlström J., Berlin M., Sköld C. M., Wigzell H., Eklund A., Grunewald J. (1999). Phenotypic analysis of lymphocytes and monocytes/macrophages in peripheral blood and bronchoalveolar lavage fluid from patients with pulmonary sarcoidosis. *Thorax*.

[25] Gogos C., Kotsaki A., Pelekanou A. (2010). Early alterations of the innate and adaptive immune statuses in sepsis according to the type of underlying infection. *Critical Care*.

[26] Steinmüller M., Srivastava M., Kuziel W. A. (2006). Endotoxin induced peritonitis elicits monocyte immigration into the lung: implications on alveolar space inflammatory responsiveness. *Respiratory Research*.

[27] Srivastava M., Jung S., Wilhelm J. (2005). The inflammatory versus constitutive trafficking of mononuclear phagocytes into the alveolar space of mice is associated with drastic changes in their gene expression profiles. *The Journal of Immunology*.

[28] Philippart F., Fitting C., Cavaillon J.-M. (2012). Lung microenvironment contributes to the resistance of alveolar macrophages to develop tolerance to endotoxin. *Critical Care Medicine*.

[29] Smith P. D., Suffredini A. F., Allen J. B., Wahl L. M., Parrillo J. E., Wahl S. M. (1994). Endotoxin administration to humans primes alveolar macrophages for increased production of inflammatory mediators. *Journal of Clinical Immunology*.

[30] Boujoukos A. J., Martich G. D., Supinski E., Suffredini A. F. (1993). Compartmentalization of the acute cytokine response in humans after intravenous endotoxin administration. *Journal of Applied Physiology*.

[31] Hoogerwerf J. J., De Vos A. F., Van't Veer C. (2010). Priming of alveolar macrophages upon instillation of lipopolysaccharide in the human lung. *American Journal of Respiratory Cell and Molecular Biology*.

[32] Maus U., Rosseau S., Knies U., Seeger W., Lohmeyer J. (1998). Expression of pro-inflammatory cytokines by flow-sorted alveolar macrophages in severe pneumonia. *European Respiratory Journal*.

[33] Schneider C., von Aulock S., Zedler S., Schinkel C., Hartung T., Faist E. (2004). Perioperative recombinant human granulocyte colony-stimulating factor (filgrastim) treatment prevents immunoinflammatory dysfunction associated with major surgery. *Annals of Surgery*.

[34] Nierhaus A., Montag B., Timmler N. (2003). Reversal of immunoparalysis by recombinant human granulocyte-macrophage colony-stimulating factor in patients with severe sepsis. *Intensive Care Medicine*.

[35] Abraham E., Nick J. A., Azam T. (2006). Peripheral blood neutrophil activation patterns are associated with pulmonary inflammatory responses to lipopolysaccharide in humans. *The Journal of Immunology*.

[36] Corvol H., Fitting C., Chadelat K. (2003). Distinct cytokine production by lung and blood neutrophils from children with cystic fibrosis. *American Journal of Physiology—Lung Cellular and Molecular Physiology*.

[37] Pang G., Ortega M., Zighang R., Reeves G., Clancy R. (1997). Autocrine modulation of IL-8 production by sputum neutrophils in chronic bronchial sepsis. *American Journal of Respiratory and Critical Care Medicine*.

[38] van den Berk J. M. M., Oldenburger R. H. J., van den Berg A. P. (1997). Low HLA-DR expression on monocytes as a prognostic marker for bacterial sepsis after liver transplantation. *Transplantation*.

[39] Monneret G., Lepape A., Voirin N. (2006). Persisting low monocyte human leukocyte antigen-DR expression predicts mortality in septic shock. *Intensive Care Medicine*.

[40] Wu J.-F., Ma J., Chen J. (2011). Changes of monocyte human leukocyte antigen-DR expression as a reliable predictor of mortality in severe sepsis. *Critical Care*.

[41] Livaditi O., Kotanidou A., Psarra A. (2006). Neutrophil CD64 expression and serum IL-8: sensitive early markers of severity and outcome in sepsis. *Cytokine*.

[42] Song S. H., Kim H. K., Park M. H., Cho H.-I. (2008). Neutrophil CD64 expression is associated with severity and prognosis of disseminated intravascular coagulation. *Thrombosis Research*.

[43] Chen Q., Shi J., Fei A., Wang F., Pan S., Wang W. (2014). Neutrophil CD64 expression is a predictor of mortality for patients in the intensive care unit. *International Journal of Clinical and Experimental Pathology*.

[44] Danikas D. D., Karakantza M., Theodorou G. L., Sakellaropoulos G. C., Gogos C. A. (2008). Prognostic value of phagocytic activity of neutrophils and monocytes in sepsis. Correlation to CD64 and CD14 antigen expression. *Clinical and Experimental Immunology*.

[45] Bae M. H., Park S. H., Park C.-J. (2015). Flow cytometric measurement of respiratory burst activity and surface expression of neutrophils for septic patient prognosis. *Cytometry Part B: Clinical Cytometry*.

[46] Hirsh M., Mahamid E., Bashenko Y., Hirsh I., Krausz M. M. (2001). Overexpression of the high-affinity Fc*γ* receptor (CD64) is associated with leukocyte dysfunction in sepsis. *Shock*.

